# Machine learning based fault classification for improved induction motor performance

**DOI:** 10.1371/journal.pone.0335367

**Published:** 2025-11-06

**Authors:** Zawar Ahmed Khan, Muhammad Amir Raza, Muhammad I. Masud, Touqeer Ahmed Jumani, Muhammad Faheem, Mohammed Aman

**Affiliations:** 1 Department of Electrical Engineering, Mehran University of Engineering and Technology SZAB Campus Khairpur Mir’s, Sindh, Pakistan; 2 Department of Electrical Engineering, College of Engineering, University of Business and Technology, Jeddah, Saudi Arabia; 3 Department of Electrical and Computer Science, College of Engineering, A’ Sharqiyah University, Ibra, Oman; 4 VTT-Technical Research Centre of Finland Ltd., Espoo, Finland; 5 Department of Industrial Engineering, College of Engineering, University of Business and Technology, Jeddah, Saudi Arabia; Wadia Institute of Himalayan Geology, INDIA

## Abstract

This study explores the design of an effective fault classification algorithm for 3 phase induction motor, an integral unit in many industrial systems. It is found that traditional fault detection methods and deep learning approaches are both effective; however, current techniques can either be computationally exhaustive, or suffer from low accuracy, thus making them inapplicable in many real-world settings. To address these limitations, this study evaluates different machine learning algorithms for accurate and efficient fault detection using a dataset of triaxial vibrational data converted into current variables. A dataset of triaxial vibrational current data at 0.7 mm bearing and rotor faults at various loads (100W, 200W, and 300W) were considered. For the data preprocessing, we handled with the missing values by interpolation and handle data imbalance fault types with Synthetic Minority Over-sampling Technique (SMOTE). Through Fast Fourier Transform (FFT) techniques, the frequency domain information were extracted, which is key for current signals, adding to the feature set. In addition, dimensionality reduction with Principal Component Analysis (PCA) and feature selection was done with SelectKBest. Then, the different machine learning models such as Random Forest (RF), Decision Tree (DT), k-nearest neighbors (KNN), and eXtreme Gradient Boosting (XGBoost) was trained to optimize the hyperparameters and make them perform to its best possible. The results shows the accuracy and performance of all models, DT and RF show good performance, with 99.95% accuracy, while KNN performs well, but at a higher computational cost in testing. Generally known for its capability to handle all the complex dataset, XGBoost wasn’t able to perform in this scenario as it got an accuracy of 87.13%, indicating potentially more optimization is required for the model. This work serves as the groundwork for future work with a multiplicity of fault types, motor specifications, and the incorporation of additional feature-engineering techniques to develop a more robust and intelligent framework for fault detection.

## 1 Introduction

Induction motors are mostly used especially in industries due to their efficiency and cheap to purchase. Yet they can encounter a range of issues that cause its functioning to cease or at least be slowed down and lead to some revenue loss [1]. These are further subdivided into mechanical as well as electrical faults based on the type of problem encountered. Mechanical fault involves bearing faults and rotor faults while electrical faults are overload, open phase and short circuit conditions [2]. These classifications will help to determine proper fault and its diagnosis as well as knowing the faults to expect when carrying out predictive maintenance [3]. In certain cases, mechanical faults are even harder to capture as compared with the electrical faults [4]. The often mechanical faults witnessed; bearing faults, rotor faults, and shaft faults. These faults engender vibration or change in the motor’s acoustic noise, and hence, vibration measurement is frequently preferred [5].

Detection of induction motor faults are very important as these machines are used mostly in industrial applications [6]. In order to preserve operational efficiency, early and precise fault detection is crucial since faults in induction motors can result in considerable downtime and maintenance expenses [7]. Several methodologies, including as deep learning, machine learning, and signal processing approaches, have been investigated to improve the precision and dependability of defect diagnostic systems [8]. Induction motor faults are diagnosed as a critical area of research because of its wide usage in industrial applications. Induction motors are susceptible to a variety of faults, downtime, and costly maintenance make early and accurate fault detection critical to an efficient operation [9]. To improve accuracy and reliability of fault diagnosis systems, different techniques of machine learning, deep learning and signal processing methods have been widely used [10].

**Fig 1:**
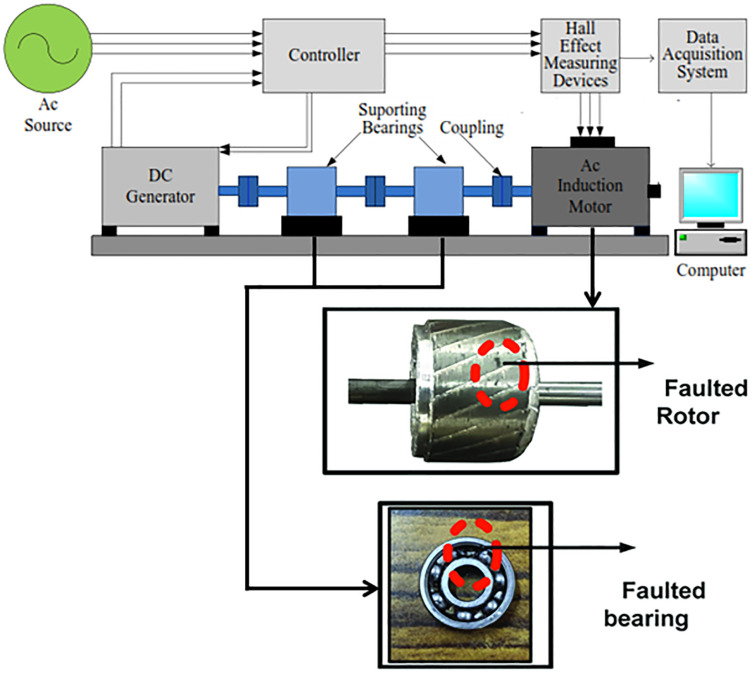
Fault classification setup [15].

Mostly, faults exist on induction motors used in power generation, manufacturing, and transportation systems under fluctuating loads [11]. The most frequently encountered defects are bearing wear, rotor bar defects, insulation breakdown, and stator winding defects, all resulting in equipment failures [12]. 12% of failures are caused by rotor faults and 51% by bearing faults [13]. Early indication of these faults was difficult to avoid systemic failure [14]. Hence this study is more focused on bearing and rotor fault detection of induction motors. However, these issues have a great impact on the motor efficiency and industrial operations, therefore calling for robust diagnostic techniques. Generally, fault classification means the identification of certain problems like inner race, outer race or broken rotor bar (BRB) fault by analyzing vibration and current to pinpoint the operational condition as shown in **Fig 1**. They are reliable and affordable, but their performance can suffer from mechanical failures and so machine learning solutions for early fault detection are needed.

This study is used to rectify the rotar and bearing faults of induction motor using the supervised learning techniques like Random Forests (RF), Decision Tree (DT), K-Nearest Neighbors (K-NN), and Extreme Gradient Boosting (XGBoost). Bearing faults are brought about by wear and tear of the bearings which act as the support structures for the rotating components of the motor. This failure can occur in two forms, inner-race faults, or outer-race faults, based on the section of the bearings that are faulty. In our case, we identify and then differentiate between faults in bearings based on the load levels which include 100W, 200W, and 300W. Further, comparision between normal working bearings and bearings with faulty conditions using the features that are vibration and current are presented. Motor bearing defects are one of the typical sources of mechanical failures, and their identification at an early state can help avoid severe damage to the motor system. Furtherm, a common type of rotor fault dubbed broken bars affects a motor’s operation when one or more bars in the rotor cage are damaged. In an induction motor the squirrel cage type of construction for the rotor includes rotor bars. A broken bar could lead to fluctuations in torque or speed and higher energy consumption at the operational level, which in turn, considerably distort efficiency at the motor. Further, the dataset is available on BRB data at two load levels like 100W and 300W for identifying and categorizing this problem with machine learning algorithms. Since failure to diagnose BRB early, the condition can lead to motor abnormalities, early diagnosis of BRB is crucial.

It is identified that, supervised learning techniques like RF, DT, K-NN, and XGBoost have been proved to be powerful tools for diagnosing motor faults through machine learning algorithms, in which vibrational and current data could be used to predict faults. Labeled data used for training these models are vibration signals linked to specific types of faults; thus these models can be trained to classify faults accurately. It’s important that feature extraction and data preprocessing improve model performance. Frequency and statistical domain features obtained by the means of Fast Fourier Transform (FFT) are also very important. To workaround the data imbalance problems, Synthetic minority oversampling technique (SMOTE) is used to synthetize instances associated with underrepresented fault classes, generating more balanced datasets. Principal Component Analysis (PCA) to reduce dimension for both improving computation and retention of the important data patterns leads to farther improved fault classification accuracy and feature selection was done with SelectKBest. In this study, both PCA and SelectKBest were intentionally applied, but for distinct purposes in the feature engineering pipeline. PCA was used as a dimensionality reduction tool, primarily to visualize the structure of the data and observe clustering patterns between different fault types. It helped to understand the variance distribution and was useful for insight into data separability in lower dimensions. However PCA transforms the original features into uncorrelated linear combinations which while useful, may not retain interpretability. On the other hand, SelectKBest was used for feature selection, retaining features with the highest statistical relationship with the target class. This ensured that only the most relevant and interpretable features such as Current-C Current-A, and crest_factor were used in the final model training. Therefore, the models were trained on the original domain features selected via SelectKBest, not on PCA components. PCA was applied exploratorily and visually, not as part of the final input to the classifiers. This allowed for a balance between: Interpretability (via SelectKBest), and Exploratory understanding of feature distribution via PCA. This separation of roles aligns with best practices where PCA is often used for data analysis and visualization, while feature selection is used for model input optimization.There is considerable evidence that combining feature extraction, data reengineering and machine learning techniques can be useful for achieving SDG 9 and SDG 12, which all highlight the importance of sustainable, efficient and innovative solutions in industrial practices.

## 2 Literature review

Faults in electrical machines are undesirable conditions which interrupt normal current or mechanical movement and may damage or break down unless identified and taken care of on time [16]. These errors are classified into general faults; open circuit and short circuit faults [17]. Open circuit faults arise when the conductors or a component opens up, stopping the current and short circuit faults arise when undesirable connections are made between phases, or between a phase and ground such that too much current flows [18]. Short circuit faults are further divided into symmetrical and unsymmetrical fault [19]. Symmetrical faults engage all the three phases at the same degree; they are quite span statistically [20]. Symmetrical faults are uncommon yet intense like three-phase faults [21]. More frequent are unsymmetrical faults that hit one or two phases in irregular fashion, such ones are single line-to-ground fault, line-to-line faults as well as double line-to-ground fault [22]. This gives rise to asymmetrical faults that result in mismatched currents in the machine resulting into stress and possible damage [23]. In addition to electrical faults, there may be mechanical faults such as failures in bearing, rotor, misalignment shaft among others or thermal fault such as overheating either of machines [24]. Electrical faults of machines specifically are short circuiting, open circuiting, insulation failures and winding faults [25]. The failures of insulation are dangerous in that they may allow unwanted current paths and cause fault currents and damages [26].

Mechanical failures in electrical machines represent an important type of breakdowns, ranked second after electrical faults in frequency [27]. These defects tend to be caused by mechanical problems in the mechanical parts of a machine like bearings, shafts and rotors [28]. Bearing failures, shaft misalignment, rotor imbalance and cooling fan damages are common mechanical failures [29]. Bearing faults that may be due to loss of lubrication, wear, or contamination results in increased vibrations, noise as well as machine down-break [30]. The misalignment of the shaft occurs when the reverse installation or thermal expansion occurs and causes too much vibration and also the wear rate of bearings increases [31]. This difference in the forces makes the rotation unbalanced, which in essence leads to vibrations and also to possible damage of the rotor and components around it [32]. In induction motors, the mechanical faults especially occur in the rotor and bearing system [33]. Under poor lubrication maintenance or conditions of overload bearing, damages can be in the form of the inner or outer raceways breaking or ball defects [34]. Failure of cooling fans also portrays a mechanical error that causes overheating, which cuts the life span of the motor by destroying windings [35]. Another severe mechanical fault is broken rotor bars, particularly in squirrel cage rotors, that causes poor performance and vibrations [36]. All these faults lead to elevated operational insecurity and less performance of electrical machines. To avoid the mechanical faults, maintenance control like correct lubrication of the bearings, shafts, couplings, being well aligned and replacing the worn parts on time should be observed [37]. Supervision of some of the early signs such as abnormal vibration, noise, and temperature rise on bearings may aid in diagnosing mechanical faults before a serious damage can be sustained [38]. They should also be handled properly and stored well in the course of transportation to prevent mechanical damages such as the bending of shafts or coupling failures [39]. It is essential to have cooling to prevent overheating by ensuring that the cooling fans duty has not been impaired [40]. These preventive measures and techniques of condition monitoring increases the reliability and lifetime of the electrical machines.

The fault diagnosis process of an electrical machine follows three main stages: fault detection, classification as well as localization [41]. The most common detection implies the observation of electric parameters including current, voltage, and temperature or vibration signals [42]. The advanced methods such as machine learning or pattern recognition can be employed to distinguish the type of faults, whereas localization identifies the faulty component within the machine [43]. Such a procedure is crucial in upkeep and prevention of disastrous equipment breakage, faults might be short-lived or permanent [44]. Transient faults are not permanent and are commonly attributed to any momentary external disturbance such as lightning or animal contact and are only cleared during power cycling [45]. There are also persistent faults that cannot be corrected immediately like the situation of underground cables or mechanical faults [46]. Both of them impact the performance of machines, but the persistence of faults is more dangerous as it may lead to long-term damages in the case they are not discovered [47].

The fault diagnostics of Induction motor is mostly used in the analysis of electrical and mechanical parameters in order to detect abnormalities that show the presence of faults. Typical signal-based techniques are Motor Current Signature Analysis (MCSA), vibration analysis and thermal imaging. MCSA identifies faults by noting deviations in the spectrum of the motor current, and can provide a specific fault location through frequency components associated with a specific fault like rotor bar breaks or stator winding problems [48]. Complementary fault diagnosis vibration signal analysis detects complementary mechanical faults, and infrared thermography records thermal abnormalities caused by electrical anomalies such as bearing wear or shorted winding [49]. Signal processing techniques may include FFT, Short-Time Fourier Transform (STFT), and Continuous Wavelet Transform (CWT) to obtain useful characteristics of raw data as part of these signal-based techniques to detect faults [50]. Machine learning and deep learning models of classification provide an overall boost to the classification of faults within induction motors, including increased accuracy and feasibility of automation [51]. Artificial Neural Networks (ANN), Support Vector Machines (SVM), and Convolutional Neural Networks (CNN) are widely used techniques of the classification of different types of faults in connection with the processed features of current, vibration, or thermal data [52]. The algorithm based on fault identification in vibration signal images (texture features extraction) and multiclass SVM showed high rates of fault identification, even in noisy conditions [53]. For simultaneous detection and identification of different faults, integrated strategies of deep learning models with MCSA have been deployed, as well. Techniques involving the use of RCNN working with Speeded-Up Robust Features (SURF) on infrared images shall offer the other platform of reliable classification in various loading conditions [54]. Fault localization on the induction motor is generally done through a close examination of the detected fault characteristics in order to determine the actual location of the fault in stator winding, rotor bars, or components of the bearing. The current or vibration signal characteristics variation in nature with the enhanced signal processing techniques and the machine learning algorithm enables the online fault diagnosis systems to identify not only the presence but also the location of fault on the basis of the severity and origin of the found anomaly [55]. The state of the art empirical mode decomposition (EMD) and zero-sequence current analysis with better visualization methods such as local symmetrized dot patterns allow faults to be localized better and more intuitively, without additional sensors [56]. It also allows precise localization of faults since thermal imaging allows the detection of specific temperature increases that are linked to particular motor elements and, in turn, allow early warning and preventive actions on this basis. Furthermore, the comparative analysis of the proposed study with the past studies is presented in **Table 1**.

**Table 1:** Comparative analysis of proposed study with the past literature.

Study	Study purpose	Model used	Accuracy	Evaluation Time
[57]	To predict the induction motors faults using machine learning algorithms	KNN	90%	Not incorporated
[58]	Model-based fault detection method for induction motors	RF	85%	Not incorporated
[59]	Developing NARX neural network for fault classification	NARX neural network	94-98%	Not incorporated
[60]	Study contributes to fault detection with machine learning for industries	DT	92%	Not incorporated
[61]	To optimize the fault detection techniques for 3-phase electric drives	Signal processing techniques	90-98%	Not incorporated
[62]	Classification assessment of induction motor	Deep learning model (EfficientNet-B0)	97%	Not incorporated
[63]	Principal component analysis and intelligent systems in three phase induction motors	Artificial Neural Network	90%	Not incorporated
[64]	Machine learning techniques for fault detection and classification	RF and KNN	98%	Not incorporated
[65]	Fault detection of induction machines	KNN	98%	Not incorporated
Our Proposed Work	Machine learning based fault classification of 3 phase induction motor	RF, DT, KNN and XGBoost	RF 99.95% DT 99.96% KNN 99.96% XGBoost 87.13%	RF 6 sec DT 0.04 sec KNN 11 sec XGBoost 3.5 sec

Hence, the applications of the proposed work encompass industries where the machines heavily rely on induction motors for their continual operation. Proactive maintenance using accurate fault detection helps reduce downtime, repair costs, and ties in with sustainable development goal 9 (Industry, Innovation, and Infrastructure) and 12 (Responsible Consumption and Production). It is identified that selected machine learning models can achieve high accuracy with much less of the computational burden required for deep learning, providing a practical and scalable approach to the fault diagnosis problem across industrial settings.

## 3 Research methodology

**Fig 2:**
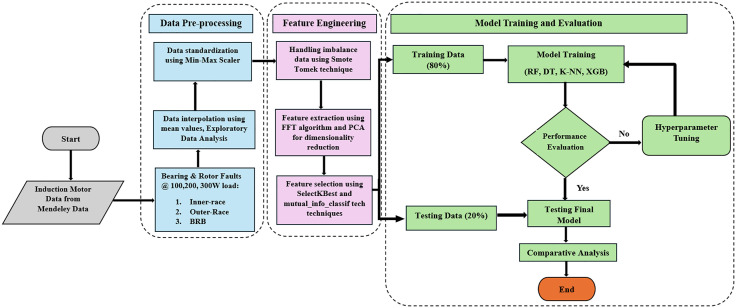
Methodology framework of proposed study.

The methodology of the proposed study is shown in **Fig 2** underlying the comprehensive development of a classification system on induction motor faults. In this research, a supervised learning framework is adopted to classify induction motor faults using triaxial vibrational data under varying operational loads via use of machine learning algorithms. It focuses on differentiating fault types such as inner race, outer race, and BRB defects to guarantee robust fault detection under a wide change in operational conditions. It covers data collection and pre-processing, feature engineering and model evaluation. The method is carefully crafted to attain computational efficiency for industrial applications while preserving accuracy of fault classification. The study focuses on developing efficient and accurate machine learning based fault classification models for three-phase induction motors, the study covers the major three domains which are discussed below:

1. To develop high-accuracy machine learning models surpassing previous benchmarks (90%−96%) by employing FFT for feature extraction and SMOTE for data balancing. This approach ensures robust fault classification across varying load conditions.
2. To compare RF, DT, KNN and XGBoost based on accuracy and computational efficiency. This analysis identifies the most suitable model for real-time predictive maintenance systems, emphasizing timely fault detection.
3. The performance measures such as accuracy, precision, recall, F1 score, and computational time. All of our machine learning models to perform fault detection under different operational circumstances will be trained from preprocessed data to enhance their flexibility and abstraction.

### 3.1 Data collection and pre-processing

The data set is taken from “Mendeley Data” entitled “Current Signature Dataset of Three-Phase Induction Motor under Varying Load Conditions” published by the professors at Mehran University of Engineering and Technology, Jamshoro [66]. The time domain dataset includes the instantaneous values of three-phase current signature data of loaded induction motor with healthy and unhealthy conditions. The motor with various faults were operated under different load conditions to observe their impact on current signatures. The motor faults include inner-race and outer-race bearing faults under varying load. Due the usage of triaxial vibrational sensors for the data acquisition the x,y,z variables were termed as Current A, B, C. These are not current values these are the triaxial vibrational data which was acquired at a sampling rate of 10 kHz at the rate of 1000 samples per channel [67]. The dataset as shown in Table 2 contains three current variables (current-A, current-B, and current-C) current at x, y and z axes respectively. The motor’s operational state under normal conditions and containing different faults are captured by these variables. Data was further reduced to 0.7 mm sized datasets, with include complete BRB data, and 0.7 mm data was restricted to the 0.7 mm dataset for all the load conditions.

**Table 2:** Raw dataset.

Sr.no	Current-A	Current-B	Current-C	Fault Type
**0**	2.4799	3.0415	2.0147	Healthy
**1**	2.4799	3.0415	2.0147	Healthy
**2**	2.4799	3.0415	2.0147	Healthy
**3**	2.4799	3.0415	2.0147	Healthy
**4**	2.3761	3.094	2.0696	Healthy
...	...	...	...	…
**1096679**	2.0794	3.1514	2.3162	Inner @ 100
**1096680**	2.0794	3.1514	2.3162	Inner @ 100
**1096681**	2.0794	3.1514	2.3162	Inner @ 100
**1096682**	2.0794	3.1514	2.3162	Inner @ 100
**1096683**	2.0794	3.1514	2.3162	Inner @ 100

First, we needed to prepare the data for analysis because data-preprocessing was a critical first step. A linear interpolation technique was considered for dealing with missing data. This method can estimate values where data is known and creates a continuous dataset which is required for machine learning model training. They also interpolated missing values in the data, and manually labeled all of the data to create ground truth available for model training in the preprocessing stage. To preserve data integrity and avoid misleading machine learning, data points with missing data were replaced with mean value interpolation. The models were able from known operational states and load conditions to label readings with applicable fault types.

### 3.2 Exploratory data analysis

**Fig 3:**
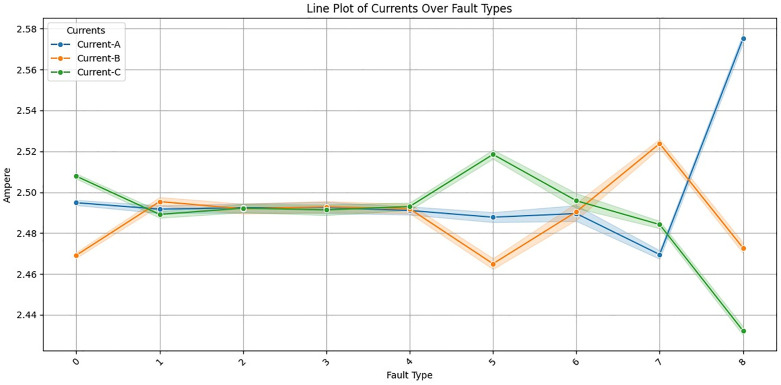
Line plots current over fault type.

To know how the data are distributed and related, we undertook an exploratory data analysis. The induction motor data pattern and correlation is analysed to find out the effect of the alteration in Current-A, Current-B, and Current-C. These all three current variables are shown in **Fig 3** which is measured in the three phases of the line plot on the x-axis and current value of the different fault types is measured in amperes on the y-axis.

**Fig 4:**
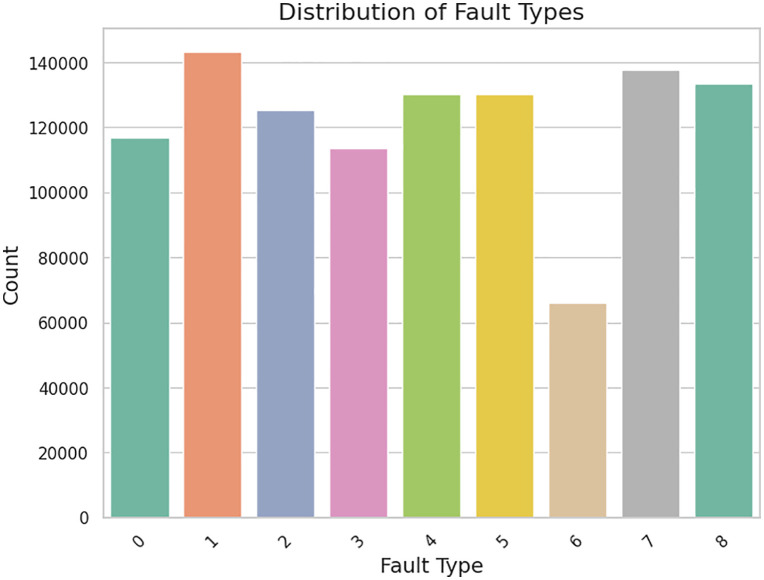
Distribution of faults types.

**Fig 5:**
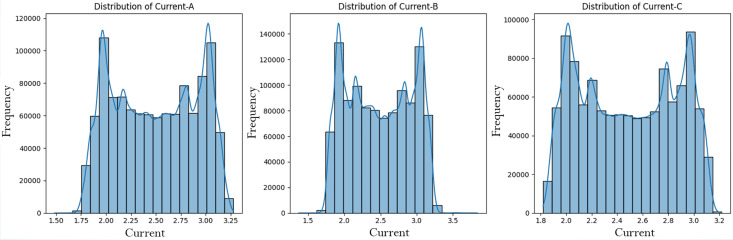
Distribution of fault currents.

Class imbalance issues such as over representation of “No Fault” cases over rare fault types were addressed using techniques such as SMOTE. The Tomek link method removes noisy samples, while SMOTE generates synthetic samples for minority classes, for a balanced clean dataset. The dataset was then explored through statistical analysis in effort to understand feature distributions, relationships, and variability as shown in **Fig 4**. The three current variables and fault types were calculated for key statistical properties including mean, standard deviation, quartiles. Insights into data diversity and variability that are critical in distinguishing real motor states from faulty ones were revealed by this analysis. Current-A, Current-B, and Current-C variations across fault types can serve as good possible candidates for providing a good classification for faults as shown in **Fig 5**.

### 3.3 Feature engineering

In feature engineering, helpful parameters were extracted from the vibrational signals to improve machine learning model performance. FFT based frequency domain analysis was an important part of this process. For this reason, FFT converts time domain signals to frequency domain components as shown in **Table 3**, which identify harmonics related to particular fault frequencies. By transforming the signal, we improve signal to noise ratios and therefore can detect suitable fault signatures.

**Table 3:** FFT feature extraction.

Sr.no	Current-A	Current-B	Current-C	mean	median	std	peak_to_peak	rms	crest_factor	FFT_mean	FFT_std
**0**	0.55566	0.67739	0.13977	0.45761	0.55566	0.230174	0.53762	0.51223	1.04957	0.78312	0.41698
**1**	0.55566	0.67739	0.13977	0.45761	0.55566	0.230174	0.53762	0.51223	1.04957	0.78312	0.41698
**2**	0.55566	0.67739	0.13977	0.45761	0.55566	0.230174	0.53762	0.51223	1.04957	0.78312	0.41698
**3**	0.55566	0.67739	0.13977	0.45761	0.55566	0.230174	0.53762	0.51223	1.04957	0.78312	0.41698
**4**	0.49762	0.6987	0.17904	0.45846	0.49762	0.21395	0.51966	0.50592	1.02716	0.76103	0.4344
...	...	...	...	...	...	...	...	...	...	...	...
**1290127**	0.56449	0.69773	0.12318	0.4618	0.56449	0.245542	0.57455	0.52302	1.09853	0.80905	0.40754
**1290128**	0.89149	0.42468	0.14413	0.48677	0.42468	0.308247	0.74735	0.57616	1.29713	0.92269	0.38014
**1290129**	0.24504	0.48364	0.80179	0.51016	0.48364	0.228064	0.55675	0.55881	0.99631	0.83269	0.49341
**1290130**	0.91743	0.30028	0.29785	0.50519	0.30028	0.291498	0.61957	0.58326	1.06227	0.91743	0.42295
**1290131**	0.56516	0.70714	0.11001	0.46077	0.56516	0.254707	0.59713	0.52649	1.13418	0.82098	0.39692

FFT application showed fault specific frequency patterns including peaks from bearing defects or rotor periodicities. To further augment these features, statistical measures such as root mean square (RMS) and crest factor were added which gave a complete representation of the data. The models gained a clearer focus on the relationships between different fault types and the current variations, resulting from focusing on frequency domain characteristics.

**Fig 6:**
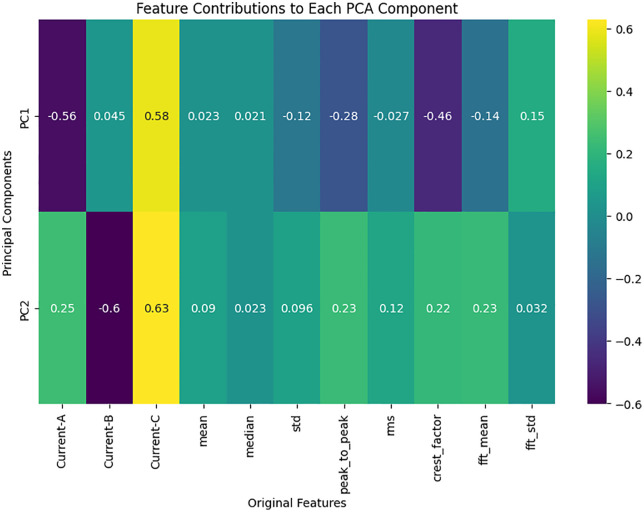
Feature contributions to each PCA component.

PCA feature selection techniques were used to optimize the dataset as shown in **Fig 6**. PCA is used by reducing dimensionality and transforming correlated feature with the most significant data variance by transforming them into uncorrelated principal components. Just like, the current-C and current-A were highly influential in explaining the data variability using PCA. Features were selected by SelectKBest on which fault classification is the best (current-C, current-A, crest factor). It improves computational efficiency and reduces error in the model, as these were streamlined techniques.

Thus, the dataset was divided into training (80%) and testing (20%) subset, in such a way that the model performance was evaluated unbiassed like maintaining independence. Four fault classification algorithms were implemented like RF, DT, KNN, and XGBoost. All algorithms provided advantages but tree based (RF and DT) were especially powerful because of their interpretability and non linearity (ability to handle non linear relationships).

### 3.4 Machine learning models

#### 3.4.1 Random forest.

**Fig 7:**
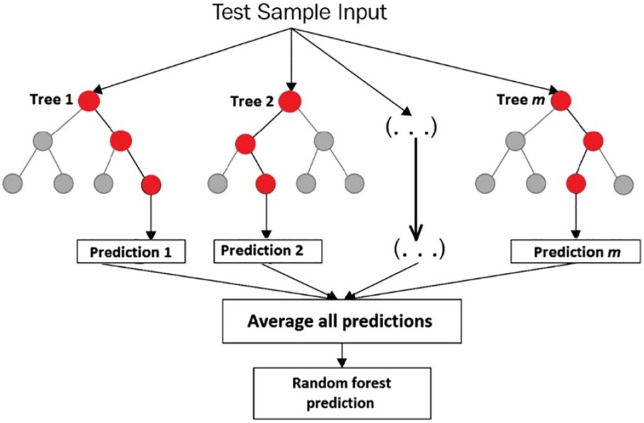
Random forest model working [70].

For classification tasks, RF is an ensemble learning technique that builds several decision trees during training and outputs in learning mode [68]. It is especially helpful in minimizing overfitting [69]. Following **Fig 7** illustrates the working of the random forest model.

Mathematically the RF classifier can be described as follows: for each tree in the forest a randomized subset of the features and data samples is chosen [71]. This subset is used to train a decision tree using the information gain or Gini impurity criteria. Calculating the Gini impurity (G) according to the Eq. (1) [72]:


$$G=\text{1}-\;\sum_{i=\text{1}}^{C}{pi}^{\text{2}}$$ (1)

where ${pi}^{\text{2}}$ is the probability of a sample belonging to class i and C is the number of classes. After all trees are trained, the classification is determined by majority voting across the trees as present in the Eq. (2) [73]:


$$\hat{yi}=mode\;\left({\hat{y}}_{\text{1}},\;{\hat{y}}_{\text{2}},\;\ldots \ldots \ldots ,\;{\hat{y}}_{n}\right)$$ (2)

where $\hat{yi}$ is the predicted class by tree i, and n is the total number of trees.

#### 3.4.2 Decision tree.

**Fig 8:**
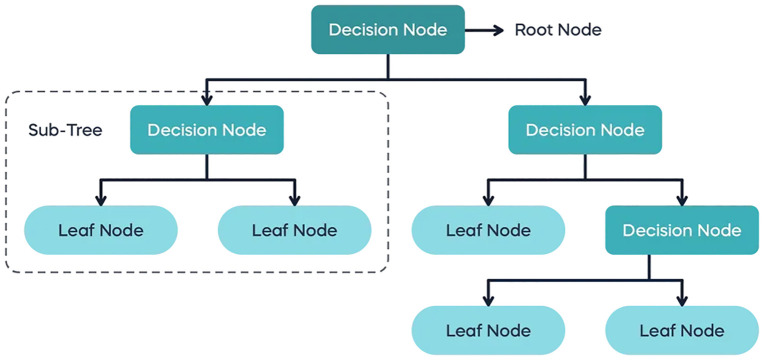
Decision tree model working [75].

A model of decisions resembling a tree as shown in **Fig 8** is produced by the DT method using input feature data. A particular feature is used to make decisions at each internal node, and a class label is represented by each leaf node [74].

Mathematically the DT classifier can be described by using Eq. (3). Entropy or Gini Impurity is used to split the nodes. The entropy H of a node is present in the Eq. (3) [76]:


$$H(S)=-\;\sum_{i=\text{1}}^{C}pi\;{log}_{\text{2}}pi$$ (3)

where S is the set of samples at a node, pi is the proportion of samples belonging to class i, and C is the number of classes. The optimal split is the one that maximizes the information gain as present in the Eq. (4) [77]:


$$IG(S,A)=H(S)-\sum_{\vartheta \in A}\frac{\left|S\vartheta \right|}{\left|S\right|}\;H(S\vartheta )$$ (4)

where A is the attribute used for splitting, and Sϑ represents the subset of S where attribute A takes value ϑ.

Decision trees are widely used due to their ease of interpretation and low processing overhead. As previously noted, their performance in this research was exceptionally strong, attaining the highest accuracy. This can be attributed to their capacity to handle non-linear relationships in the feature set [78].

#### 3.4.3 K-Nearest neighbor.

**Fig 9:**
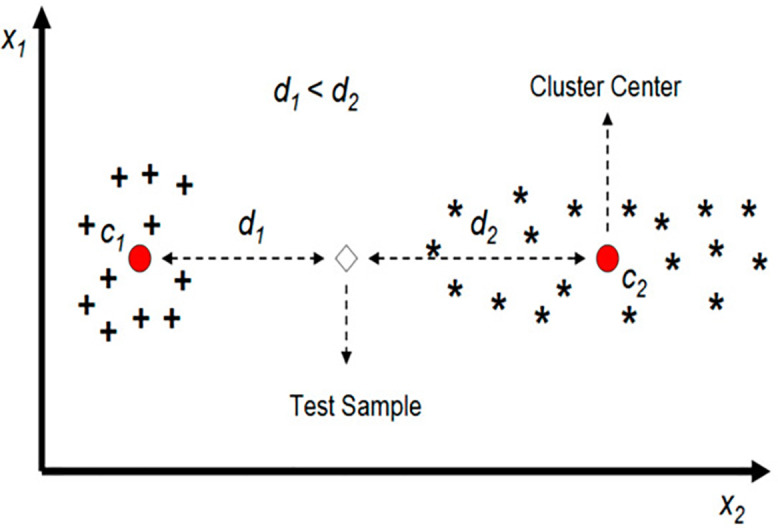
KNN model working [80].


KNN is an instance-based, non-parametric learning technique that determines a test sample’s class by looking at its KNN majority class in the feature space as shown in **Fig 9**. In KNN, the distance between data points, typically measured using euclidean distance, which is present in the Eq. (5) [79]:

$$d\left(x_{i},\;x_{j}\right)=\;\sqrt{\sum_{k=\text{1}}^{n}\left(x_{i},k-\;x_{j},\;k\right)}$$ (5)

where $x_{i}\;$and $x_{j}$ are the feature vectors of the test sample and training sample, respectively, and n is the number of features.

When the number of features rises, KNN suffers from the curse of dimensionality, although being extremely effective in low-dimensional settings [81]. Its performance in this research was significantly poor, partly because of the altered data following feature extraction had more dimensions [82].

#### 3.4.4 Extreme gradient boosting.

**Fig 10:**
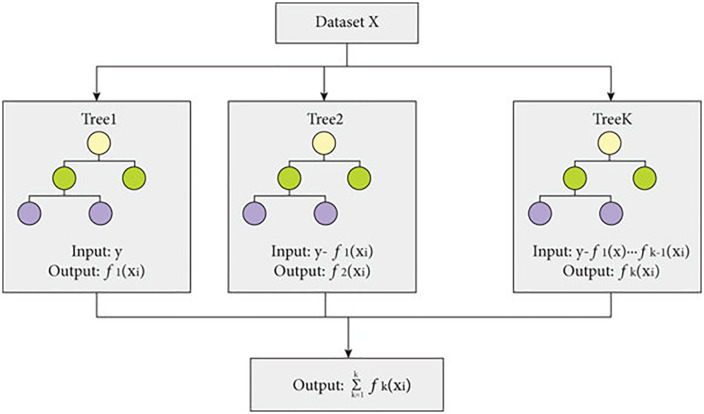
XGBoost model working [86].


With decision trees acting as weak learners, XGBoost is a robust gradient boosting method. Following **Fig 10** shows the working of XGBoost model working for fault classification. By using gradient descent to minimize a differentiable loss function, it improves model performance. The prediction function in XGBoost is present in the Eq. (6) [83]:

$$\hat{y}i=\;\sum_{k=\text{1}}^{K}fk\;\left(x_{i}\right)$$ (6)

where fk is the k-th weak learner (a decision tree), and K is the total number of trees. The objective function is defined in the Eq. (7) [84]:


$$L=\;\sum_{i=\text{1}}^{n}l\left(\hat{y}i,\;yi\right)+\;\sum_{k=\text{1}}^{K}\Omega (fk)\;$$ (7)

where $l\;(\hat{y}i,\;yi)$ is the loss function, and Ω(fk) is a regularization term to penalize the complexity of the model. The gradient and hessian of the loss function are computed to optimize the weights of the trees as present in the Eq. (8) [85]:


$$g_{i}=\;\frac{\partial l\left(\hat{y}i,\;yi\right)}{\partial \hat{y}i\;}\;\;,\;\;\;\;h_{i}=\;\frac{\partial^{\text{2}}l\left(\hat{y}i,\;yi\right)}{\partial \hat{y}i^{\text{2}}\;}$$ (8)

XGBoost is well-known for its efficiency and speed, especially when working with large data sets. However, because the fault signals in the present research were complicated and non-linear, XGBoost did not perform as well [59].

Metrics for model performance were evaluated: accuray, precision, recall, F1-score, and inference time. The accuracy was the proportion of correct predictions, the precision and recall were the measures of the model’s capabilities to correctly predict a type of fault including false positives and false negatives [87]. A balanced analysis of precision and recall using the F1 score is very important for the imbalanced dataset. The speed at which models generated predictions was quantified in terms of inference time critical for real time applications.

In conclusion, this methodology is suggested as a systematic way to create machine learning models of induction motor fault classification. The study achieves robust fault detection with high accuracy and computational efficiency by addressing data quality, feature engineering and model optimization. This research yields insights that enable the design of scalable, real time fault diagnosis systems to support predictive maintenance and industrial operational reliability.

## 4 Results and discussion

The results elaborate on the understanding gleaned from the methodology to explain how the feature extraction, feature selection, data standardization, and machine learning modeling have resulted in the outcomes of study. Further it is summarized that why the models were used, the significance of the features included in the models and the deconstructing of the outcomes from the methodology employed. Hence it is proceeded with the scale that have range of features with Min Max scaling on our processed dataset and presented it as it data input. It was able to shield the computation of our machine learning model from severe transformations from the input variable (due to distance), thus allowing it to work correctly at the level of high performance. Another scaling was performed of these statistical and signal-based features: mean, median, rms and crest factor with 3 other 3 features; current-A, current-B and current-C in the current modelling which then had to be correlated with each other. However, as FFT feature extraction has proved to be very useful in improving dataset since it consists of time domain current signals now transformed into frequency domain. Therefore, with this modification it was possible to capture periodic variations, as well as more important signal patterns for the detection of different types of induction motor faults. Since the current signals break down existing signals into their individual frequencies, we were able to detect smaller oscillations of motor activity that would have been lost if just analyzing the information in the time domain. In particular, statistical characteristics made it that the model had rich and informative feature collection for grouping and classification when combined with them. Select KBest, this method further improved this procedure by an attempt at using only those features that are most relevant on the dataset. This only strengthens the high relevance between current-A, current-B, current-C, and crest factor. In addition, we showed that these features were responsible for most of the motor behavior differences and were beneficial to simplify the data dimension, and retain high predictive power using a subset of models that removed some of these features from the model. In particular, this hand selected approach prevented both the noise in the fault classification being amplified and also the general stability of the classification in relation to the various types of faults. Then the variation resulting from various combination of features can be seen and studied by the PCA. Using PCA, we were able to vastly simplify the high dimension features space, and hence make the model’s decision making more intuitive. The most variation was in the first main component from which current-A, current-B, current-C contributed substantially while crest factor contributed substantially to the second component which was made abundantly evident from the PCA heatmap. As this visualization verified, the SelectKBest actually chose the most informative aspects.

### 4.1 Random forest

**Fig 11:**
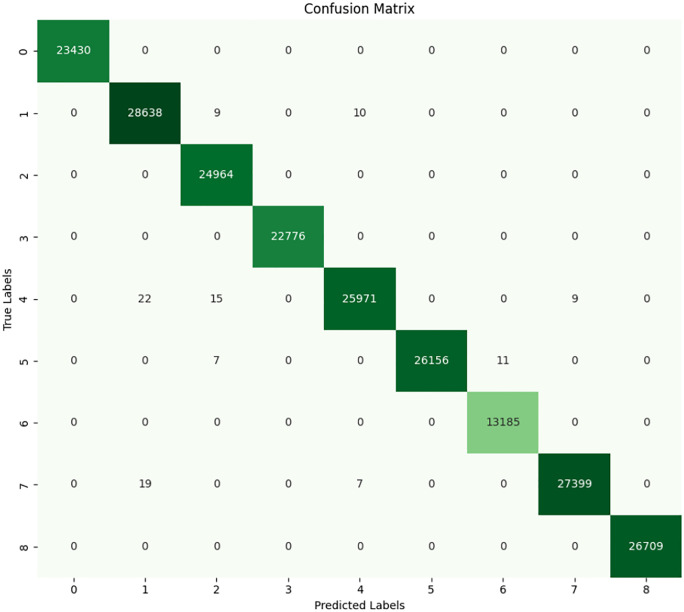
Confusion matrix of RF.

**Fig 12:**
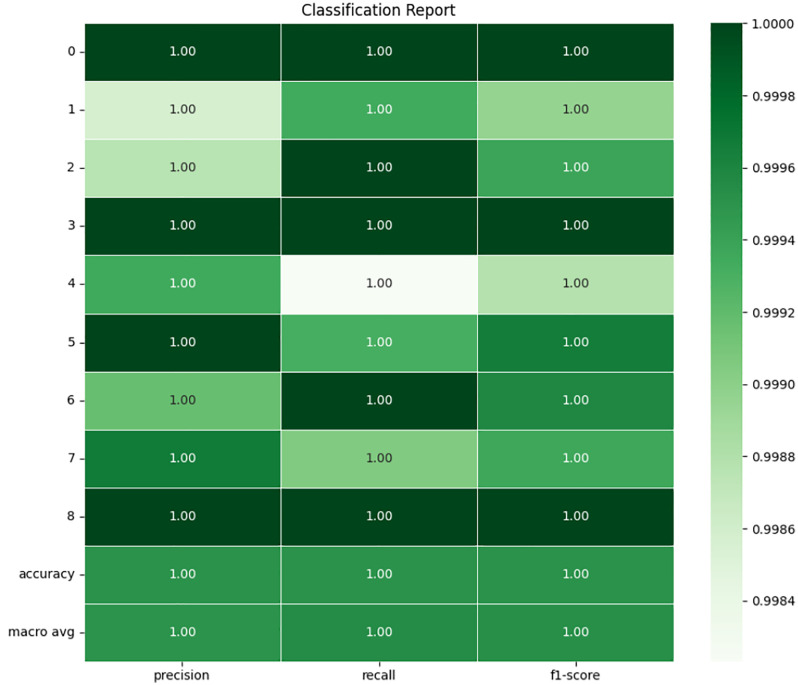
Classification report of random forest.

From the data presented by confusion matrix and classification report in **Figs 11** and **12** of RF, it is found that all performances were exceptionally high, especially for the testing set. The confusion matrix clearly shows that the RF model makes almost no misclassifications. For each fault type, the majority of the true labels align perfectly with the predicted labels. There are only a few instances of misclassification, like the model confused a few samples of Fault 4 with Fault 5 and similarly between Fault 6 and Fault 7, but these are incredibly rare. This suggests that the RF model captures the underlying patterns of the data remarkably well, particularly for the different fault types, such as bearing and rotor faults at varying loads.

Under the Fault Type 0 (No Fault), the model perfectly classifies all of the samples with no error. In case of all 70,207 samples, the model did not identify any kind of fault, which indicates that the model is very good at identifying normal operation. Under the Fault Type 7 (Broken Rotor Bar at 100W Load), the RF model performed slightly worse. It is identified that 53 cases where samples of Fault 7 were incorrectly classified, considering the fact that it still performed exceptionally effectively. This mistake percentage is still quite low, however considering the high number of samples (82,504) that were successfully identified.

The classification report displays nearly optimal metrics every class has an accuracy, precision, recall, and F1 score of 1.00 low differences in Fault Type 7, where the F1 score dips down to 0.99. Usually, when the RF model is applied to the training set of data, it is certain that it has captured detail and variations in the data accurately. The accuracy, recall, and F1 score values of more than 0.90 for each fault type clearly reveals that RF has no problem in working on the multi class features. Slightly lower classification accuracy in Fault 7 could also point to certain similarities between Fault Types 6 and 7, perhaps because of the nature of the rotor faults under varying loads, but the effect of classification error is negligible. Also, since RF is a model that performs well with large features and selecting features that do not overfit, it is ideal for our dataset that features a variety of features extracted from the current signals. Due to the model’s high performance, it is clear that these extracted features (including current signals and statistical measures) are utilized in the differentiation between various motor conditions even though the faults intersect slightly.

RF is identified as the most effective classifier for dealing with the problem. The least misclassification rates jointly with high accuracy rates presented in the classification report ensure feasible application in real-life fault diagnosis of 3-phase induction motors. Nevertheless, it remains essential to see how well these results generalize to other datasets than the training data in order to avoid that this successful performance only emerged because the algorithms overfit on the training datasets. The results of this analysis indicate that the choice of the RF model is justified, offering confident, consistent classification for all types of faults and reiterating its effectiveness as a method for fault diagnosis in industrial systems.

### 4.2 Decision tree

**Fig 13:**
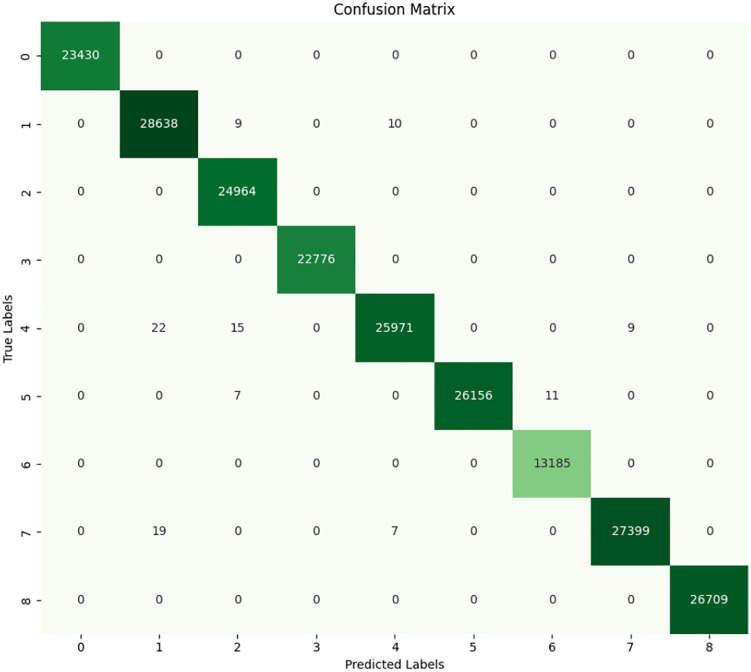
Confusion matrix of DT.

**Fig 14:**
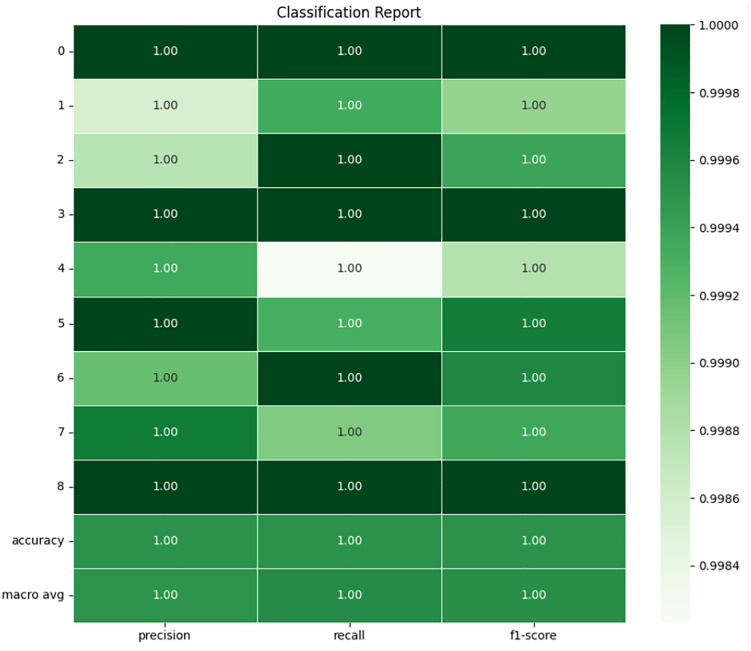
Classification report of decision tree.

The accuracy and efficiency of the DT model is evident from the confusion matrix and classification report as shown in **Figs 13** and **14** of the training and set data carrying out the fault classification with high reliability. Moreover, the identified perfect accuracy of 99.95% of the proposed model on the test set, as well as the F1-score, precision, and recall of 0.9995, confirm the almost flawless ability of the model to classify all types of faults. The analysis of confusion matrix shows that for nearly all of the various fault types the true positive values (values along the diagonal) greatly outnumbers the rest of the fault instances which shows that the model is doing a very good job identifying most of instances of the various faults. For instance, fault type “INNER at 100W (1)” is correctly classified 28, 638 instances and the total instances of 28,647 few of them are misclassified. This behavior is reflected in other fault types as well as more complex faults, such as the BRB at 100W (7), where the misclassification was only in 19 out of the total of 13,185 instances.

The classification report of offers further understanding of the performance of the DT model. For each of the classes, not only precision, but also recall and F1-scores are 1.00, which means that the model remains highly accurate in flagging faults while offering a very low risk of false positives. The macro average and weighted average also stood at 1.00, which demonstrates that the model can work with imbalanced classes effortlessly. Perhaps one of the primary advantages of the DT is its ability to provide an easy interpretation of the resulting decision-making process. This model comes into operation through successive partitioning of the data in accordance with the feature values which best minimize the impurity. Considering the type of data, we have and the fault classes, it is possible that current-A, current-B, and current-C which are triaxial current data and crest factor have been instrumental in these splits. The results demonstrating the ability of the model to identify the faults under different load conditions, for instance, INNER at 300W and OUTER at 100W also provide evidence for the model’s versatility in coping with different operating states. Indeed, the performance of the DT model in fault classification based on this particular dataset can be considered satisfactory to a high degree. The overall accuracy and recall it achieves on all defined fault categories make it ready to assist in real-time fault diagnosis for three-phase induction motors.

### 4.3 K-Nearest neighbor

**Fig 15:**
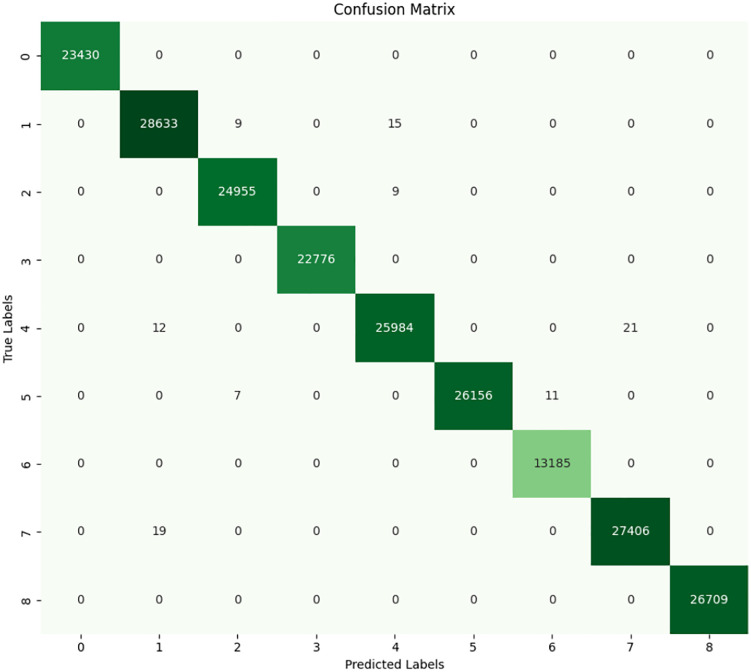
Confusion matrix of KNN.

**Fig 16:**
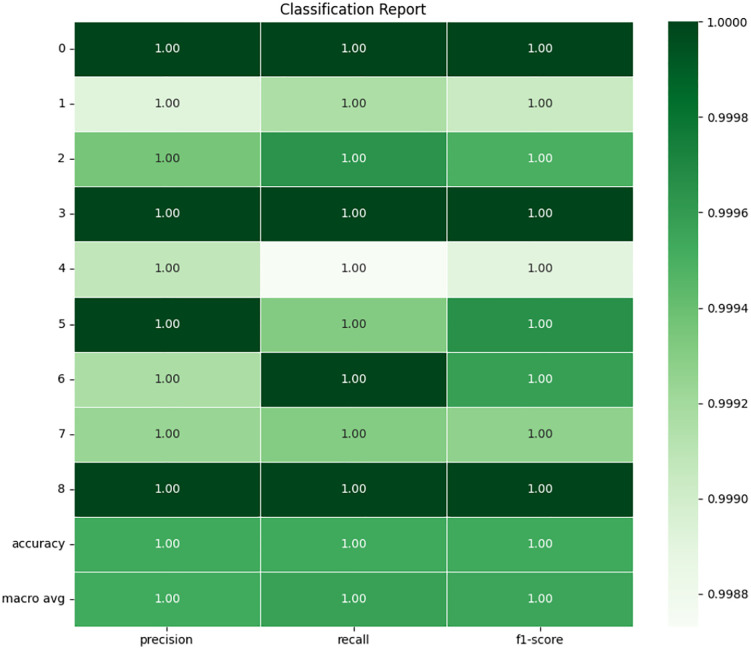
Classification report of KNN.

The KNN model is a learning model that does not impose parameters on the independence of the sample to make a classification, but rather classifies the model based on the closeness of data-points to one another in feature space. An upper stopper outperforming the KNN model for classifying faults. The overall confusion matrix and the classification report as shown in **Figs 15** and **16** reveal very high metrics across all ratings of each fault class. On the test set the model yields 99.95% accuracy which proofs that KNN algorithm is highly resilient for this specific data set. In confusion matrix, every true label is accurately matched with predicted label and there are not many chances of wrong classification. Numbers 23,430, 28,638, and 26,709 as the largest diagonal elements of the confusion matrix show the number of accurate predictions of the classes 0 (No Fault), 1 (Inner at 100W Load), and 8 (BRB at 300W Load), respectively. Peculiarities of the minimal off-diagonal values are identified in such a sense that it is still possible to allow for misclassification of a few samples belonging to classes 3 and 4, which indicates that there might be some cases where the proximity-based approach may fare marginally worse. Like, class 4 (Outer at 200W Load) exhibited 21 misclassified cases that was predicted as class 3 (Inner at 300W Load). This latter slight misclassification could be because of similar current or vibrational resonance pattern of faults that are similar in loading and position.

In classification report, it is indeed indicated that the proposed work has achieved perfect precision, as well as recall and F1-scores of one for most classes. The macro average and accuracy are quite similar, each being slightly greater than 1.00 which reflects the fact that the KNN model performs the same regardless of the specific type of fault encountered. Such execution of the model ensures that there are no false negatives, or in other words there is a perfect recall or the precision is 100%. The main advantage that is found in KNN is the ability to work with distance measurements between two given pieces of data; this makes the model versatile since it simplifies a multi-class categorization problem of different fault types that result in distinct current signal patterns. However, the reader must note that the effectiveness of KNN depends on which value is assigned to ‘k’ the number of nearest neighbors used in classification and that for the present model, clearly a good value of ‘k’ was used during model selection possibly employing cross-validation. Furthermore, normalization perhaps could have improved on the classification between the classes since K-NN is very sensitive to the scale of the features.

It is concluded that the classification performance determined by the KNN model yields nearly zero error rate for the fault detection problem with just a slightly offset that could have come from the overlapping feature space of the fault types. Because of both a high accuracy, and almost achieved best value of F1 and precision-recall curve, KNN seems to be viable for the real-world application of the fault detection in 3-phase induction motors with the given feature space.

### 4.4 Extreme gradient boosting

**Fig 17:**
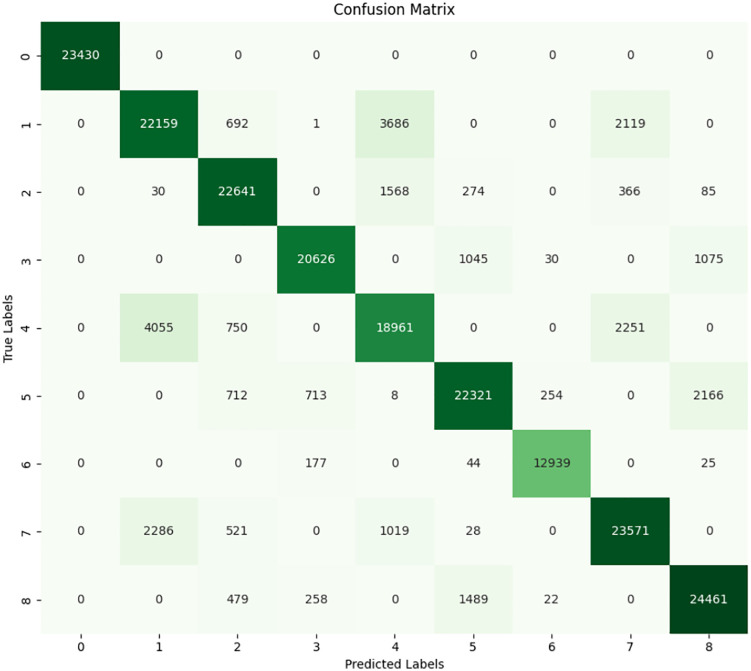
Confusion matrix of XGBoost.

**Fig 18:**
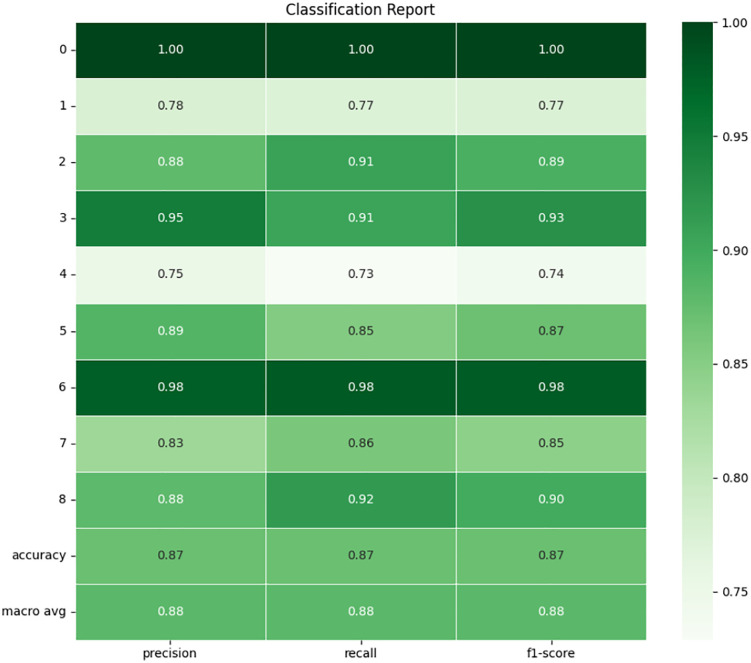
Classification report of XGBoost.

XGBoost model shows a much lower performance than DT model. The confusion matrix and and classification report of the model is shown in **Figs 17** and **18** which delivers an accuracy result of 87.13% in the test set, which is much lower compared to other models. Although this is also a relatively high accuracy, it demonstrates that the model achieves a lower accuracy in classifying specific fault types. The final F1 score is 0.8711, and it means that the accuracy of the algorithm in finding classes is of equal importance as the number of true positives towards each of the classes. It is also pointed that particular classes are not being predicted with adequate confidence level and thus results in more misclassifications. Precision and recall are equally 0.8713, which makes the model to produce frequent misclassification of both positive and negative cases concerning particular labels.

The off-diagonal entries reflect the points that have been classified wrong during sorting and clustering. The following error types are observable which buttress the fact that the model is likely to be missing some accuracy in classification of fault type like there was 4055 samples of fault type 4 (Outer at 100W load) wrongly predicted as fault type 0 (No Fault). We also have 2,286 samples with fault type 7 (BRB at 100W load) classified as fault type 0 misclassified. Furthermore, fault type 4 has 3,686 samples classified as fault type 1 (Inner at 100W load), which translates to the conclusion that the model cannot differentiate specific load conditions. Talking about the classification report of concerning the Fault type 0 (No Fault), both precision and recall reach the highest value of 1.00, which means that the model offers good results at saying that there is no fault. While all fault types are well-classified, they still have serious misclassification rates given that their precisions and recalls are low, particularly for type 1 (Inner at 100W load), which reaches only 0.77. This implies that the model tends to confuse this with any other class according to the analysis. For the other fault types, Inner at 200W and Inner at 300W load, the precision is somewhat better, 0.88 and 0.91, but recall reduces to 0.89 and 0.93, respectively. This disproportion reveals that there are some situations where these types of faults were classified as other types inappropriately. In fault type 4, which is the outer load at 100W, the precision score is 0.73 and recall of 0.74 which implies nearly 27% of cases are misclassified. Macro average of precision and recall both stand at 0.88, and this is somewhat in contradiction with the findings on the accuracy since the model’s ability to generalize is systematically low in all classes.

The model has some problems with some types of faults, which are not easily distinguishable from one another mainly because they are are similar. The distinction between faults 1 and 4 which are Inner and Outer race faults respectively at 100W load is very striking. Of these classes, there is a highly correlated prediction of each other. There is a considerable rate of misclassification errors for fault type 4 (Outer at 100W load) and fault type 7 (BRB at 100W load). Taken in the practical sense, these misclassifications are likely to result in wrong maintenance decisions, thereby compromising on the model.

After verifying the workflow, SMOTE was correctly restricted to the training data, thereby preventing any data leakage. Results without SMOTE showed noticeably lower recall and F1-scores, especially for the minority class, due to class imbalance. Introducing SMOTE increased the number of minority class samples in the training set; for example, if the original training set contained 500 minority samples and 5,000 majority samples, SMOTE would add approximately 4,500 synthetic minority instances, balancing both classes at 5,000 each in the training set. This resulted in a significant improvement in metrics. However, even with proper procedures, extremely high performance values (close to 1.00) may indicate either a trivially easy classification task or perhaps an underlying issue with the data split or feature set that warrants further inspection. The complete understanding of the proposed models’ performance is presented in **Table 4** of models implied provided by the training time, testing time, train accuracy and test accuracy. Each model have heighest testing accuracy and time taken (evaluation time) for the detection of faults in 3 phase induction motor.

**Table 4:** Comparative analysis of the proposed machine learning models.

Model Name	Accuracy (%)	Evaluation Time (seconds)
Random Forest	99.95	6
XG Boost	87.13	3.5
K-NN	99.96	11
Decision Tree	99.96	0.04

The discussion section needs to be expanded to more thoroughly analyze the results

Furthermore, the applications of machine learnings are also extended to the fault detection in helicopters [88], large electric ships [89], railways [90] and also valid for the large nuclear power complexes [91]. Alongside, the safety margin would also be increased by implementing the machine learing in transportation systems [92].

## 5 Study limitations

Few of the main limitations related to optimize machine learning based fault classification on induction motors are:

1. Huge and properly labeled datasets representing a wide range of different faults and operating conditions are prerequisites of machine learning models.
2. Motor signals that are experienced in real-life are usually noisy and variable due to change in loads [93].
3. Deep learning models are inherently accurate but usually lack interpretability, and therefore the maintenance engineers cannot comprehend the reasoning of fault predictions making it limiting to trust and use them.
4. Most machine learning fault classification approaches deliver promising results relative to laboratory-based tests, but achieve little scalabilities and adapts in other motor types or industrial settings.

## 6 Conclusion and future work

In this research, advanced machine learning techniques for fault classification of 3-phase induction motors are proposed with the objective of fault diagnosis of bearing as well as rotor faults. Industrial operations depend on induction motors, which often equates to downtime and expense. Although traditional fault detection methods already do provide an effective solution to some extent, they need human intervention, are time consuming and suffer from accuracy and generalizability limitations with respect to different load conditions and fault types. Hence, this study solved the problem using machine learning algorithms to integrate into an automated fault classifying machine and improve the prediction accuracy. A dataset of triaxial vibrational current data at 0.7 mm bearing and rotor faults at various loads (100W, 200W, and 300W) were considered. For the data preprocessing, we handled with the missing values by interpolation and handle data imbalance fault types with SMOTE. Through FFT techniques we were able to extract the frequency domain information, which is key for current signals, adding to the feature set. In addition, we performed dimensionality reduction with PCA and feature selection with SelectKBest, reducing features to only the most relevant and compressing the model’s computational time. Then, we trained a set of different machine learning models such as RF, DT, KNN, and using XGBoost to optimize the hyperparameters and make them perform to its best possible. The results shows the accuracy and performance of all models, DT and RF show good performance, with 99.95% accuracy, while KNN performs well, but at a higher computational cost in testing. Generally known for its capability to handle all the complex dataset, XGBoost wasn’t able to perform in this scenario as it got an accuracy of 87.13%, indicating potentially more optimization is required for the model. The results illustrate that 3-phase induction motor driven sector should reliant on machine learning for predictive maintenance systems will experience reduced downtime and be more efficient operationally.
